# Family members’ experiences of caring for a relative with substance-induced psychosis disorder

**DOI:** 10.4102/curationis.v45i1.2348

**Published:** 2022-11-21

**Authors:** Sanny Selotole, Annie Temane, Marie Poggenpoel

**Affiliations:** 1 Department of Nursing, Faculty of Health Sciences, University of Johannesburg, Doornfontein, South Africa

**Keywords:** caring, experiences, family member, relative and substance-induced psychosis disorder

## Abstract

**Background:**

When there is a lack of resources in the community to support deinstitutionalisation, family members of a relative diagnosed with substance-induced psychosis disorder (SIPD) are the most affected and vulnerable. Nevertheless, family members’ care is still largely unacknowledged in the mental health sector in low- and middle-income countries. Furthermore, no prior research could be found on family members’ experiences caring for a relative with SIPD in Giyani, Limpopo province, South Africa.

**Objectives:**

To explore and describe family members’ experiences caring for a relative with SIPD.

**Method:**

The study employed a qualitative research design using interpretative phenomenological analysis as the research method. Telephonic interviews were conducted and analysed. Eight family members were selected to participate in the study using a purposive sampling technique.

**Results:**

The analysis of data led to the emergence of the following themes: family members experienced caring for a relative with SIPD as a destabilising responsibility; they experienced acceptance and support from significant others and the community and solace in prayer. Participants also expressed they experienced a need for support from government structures in order to care for a relative with SIPD.

**Conclusion:**

The study’s findings highlighted the family members’ experiences of caring for a relative with SIPD and the role of the family, community and government structures in caring for an individual with SIPD. It is evident from the challenges experienced that the family members need external interventions to develop healthy coping strategies.

**Contribution:**

This study adds knowledge to nursing practice, nursing education and nursing research by promoting effective coping amongst family members caring for a relative with SIPD.

## Introduction

Substance abuse is a significant problem contributing to individuals’ mental illness. It affects the individuals abusing substances and their family, friends, the community and the healthcare system. Many individuals diagnosed with substance-induced psychosis disorder (SIPD) live in close contact with informal caregivers, typically close relatives such as their parents, siblings or children, who find it very difficult to manage the situation. People abusing substances show unpredictable behaviour and often have physical and emotional problems, including depression and aggression (Hlungwani et al. 2020:2). Many substances cause forms of intoxication and alter the individual’s judgement and perception. These individuals are frequently irritable and aggressive towards their caregivers, and their caregivers feel hopeless, frustrated and helpless when their relative relapses under their watch (Tylor 2018:17). Moreover, SIPD may cause a variety of psychosocial problems, such as decreased quality of life, for the affected individual’s family members and the person abusing substances.

Preston and Epstein (2019:53) claim some progress has been made in South Africa in terms of decentralising care for severe SIPD. However, there are insufficient resources to adequately support community-based services, resulting in the classic ‘revolving door’ phenomenon. Consequently, according to evidence from developing countries, patients with SIPD often live with their relatives in their respective homes (World Health Organization [WHO] 2020:48). According to the American Psychiatric Association (2018:141), statistics confirm that most families caring for a relative diagnosed with SIPD are not coping as a result of a lack of knowledge. In addition, some families do not value the health system.

A study conducted in the United States in the Eighties discovered that between one-third and two-thirds of persons with SIPD were cared for by their family members in their respective homes. A comparative study from China also determined that more than 50% of SIPD patients lived with their relatives, and they were unmanageable. Another United Kingdom study conducted in the Eighties confirmed that psychotic disorders such as schizophrenia, SIPD and bipolar mood disorder were severe and disabling mental health conditions that affected several million people worldwide.

The Ghanaian Health Statistics of 2014–2018 reported that 35% of Ghanaian households had at least one person with SIPD being cared for by relatives at home. This statement was supported by Ntescu (2019:245), who further claimed that SIPD patients often consulted traditional healers; their reasons for not attending health facilities remain unclear. Most of these family members viewed SIPD not as a disease but as a curse.

In South Africa, 12% of youth experiment with alcohol and cigarettes before the age of 13 years, and this behaviour progresses to substance misuse and addiction (Smith, Estefan & Caine 2018:333). There are also countrywide reports of increased substance intake amongst adolescents and middle-aged individuals in Limpopo province. Many types of substances are being abused and accessible in the community, classified as depressants, stimulants, opioids, hallucinogens and cannabinoids (Ntescu 2019:245).

While Middleton (2020:129) claims family intervention is the best therapeutic milieu for the treatment and rehabilitation of patients living with SIPD after discharge from the hospital, Prestley, McPherson and Davies (2018:123) reported that family members caring for a relative with SIPD required professional support for their own mental healthcare needs and well-being. According to Steward et al. (2015:1221), amongst carers of people with SIPD, approximately 40% report poor psychological functioning, including depression and other stress-related disorders. Cherek (2020:331) also stated that if relatives relapse, everyone at home is devastated and emotionally unbalanced. Moreover, Stuart et al. (2014:333) confirmed that family members caring for a relative with SIPD had insufficient knowledge of caring for their relatives – especially when they relapsed – therefore, professional intervention is required in order to assist them to cope. Psychiatric nurses must therefore ensure that caregiving knowledge is provided to carers through primary healthcare.

Besides the study of Hlungwani et al. (2020:1) conducted in Giyani, Limpopo province, there is a paucity of literature regarding the family members’ experiences of caring for a relative with SIPD, in Giyani, Limpopo province, South Africa. Thus, a qualitative study on family members’ experiences caring for a relative with SIPD will help to address gaps in the literature. Therefore, this study aimed to explore and describe family members’ experiences caring for a relative with SIPD. The family members’ involvement in the study is important in order to understand the family members’ experience in caring for a relative with SIPD. The research question that arose from this problem statement is as follows: what are the family members’ experiences of caring for a relative with SIPD in Giyani, Limpopo province?

## Aims and objectives

The purpose of this study was to explore and describe family members’ experiences caring for a relative with SIPD in Giyani, Limpopo province, South Africa.

## Research design and method

### Research design

A qualitative, exploratory, descriptive and contextual research design was applied (Creswell & Creswell 2018:190), using interpretative phenomenological analysis as the research method. This approach assisted the researcher in exploring family members’ experiences caring for a relative with SIPD.

### Research setting

The study took place in Giyani, one of the municipalities under the Mopani district municipality of the Limpopo province. Giyani is characterised by high levels of poverty and unemployment and low levels of education. Nearly 40% of the population (39.4%) falls below the poverty line, and 13.2% of households have no income (WHO 2020:222). In the area, large families typically live together and care for relatives abusing substances who are discharged from hospitals. They rely on the government for survival. The research setting was the homes of family members living with a relative diagnosed with SIPD in Giyani, Limpopo province.

### Population and sample

In this study, the population was family members caring for a relative with SIPD. A purposive sampling method was used to select the participants. Purposive sampling, as highlighted by Brink, Van Der Walt and Van Rensburg (2018:25), was used to select eight family members caring for a relative with SIPD. Purposive sampling also refers to ‘judgemental’ sampling since it entails making a judgement about the population to be studied (Gray, Grove & Sutherland 2017:345). The sample size was determined by data saturation, when no new information was attained and there was redundancy in information. This population was accessible to the researcher, and because of coronavirus disease 2019 (COVID-19) regulations, participants were accessed telephonically.

The sampling criteria included all family members caring for a relative with SIPD living with them at home. Participants met the following inclusion criteria:

- Participants could either be male or female.
- Participants had to be 18 years or older.
- Participants had to be able to communicate either in English or Sepedi.
- Participants had to be living with a relative diagnosed with SIPD and caring for them over a period of 5 years.
- Participants had to have a telephone, cell phone or landline.
- Relatives with SIPD had to have been admitted to a psychiatric hospital.

### Data collection

Because of COVID-19 regulations, the researcher conducted in-depth individual telephonic interviews, focusing on exploring family members’ experiences caring for a relative with SIPD. The interviews were facilitated through open-ended questions (Gray et al. 2017:344). The researcher made his own personal field notes on his own reactions, reflections and experiences under this phenomenon. The interviews’ dates, times and places were confirmed with participants and conducted between October 2020 and November 2020. During interviews, the researcher used probing to encourage a detailed exploration of family members’ experiences caring for a relative with SIPD. The researcher also employed bracketing to avoid bias in the study (Gray et al. 2017:156). Each interview lasted 40–60 min. Personal field notes were also taken during data collection, using audio recordings, a cell phone and a pocket-sized notebook.

Data were collected until saturation was achieved at the fifth participant, when the same themes emerged repeatedly. Three additional interviews were conducted to confirm the themes. The central question posed to the participants was: *what is it like to have a relative with SIPD?* Most of the interviews were conducted in Sepedi since participants did not understand English. Individual interviews were audio-recorded with permission from participants and later transcribed and translated into English.

### Data analysis

Data analysis occurred concurrently with data collection, because the researcher reflected on the raw data available in order to analyse the qualitative data. The purpose of data analysis was to make sense of all collected data by placing it in a useable format to distinguish the content (Burns & Grove 2021:502). The researcher used Tesch’s open coding method of thematic data analysis (Creswell & Creswell 2018:190) to analyse and make sense of the collected data. Codes were identified from the transcribed data and combined to form themes and supporting categories. The raw data were also given to an independent coder for analysis, purposely selected because of their experience in qualitative research. The independent coder used the same protocol for data analysis, separate from the researcher. Because of COVID-19 restrictions, the researcher and independent coder had a telephonic discussion to reach a consensus on the findings.

The principle of autonomy was respected by treating participants as autonomous agents with the freedom to conduct their lives as they chose without external control. To ensure the principle of beneficence, the researcher was sensitive to any signs of emotional discomfort from participants and scheduled individual emotional support as needed. The researcher asked appropriate questions to avoid any discomfort that could have occurred throughout the interviews.

In order to ensure the principle of justice, the researcher guaranteed that the participants’ rights to privacy, fair selection, anonymity and confidentiality, fair treatment and protection from discomfort and harm were adhered to throughout the study. The researcher also excluded all social, cultural, racial and sexual biases (Gerrish & Lacey 2016:112). In addition, written informed consent was obtained from participants prior to conducting the telephonic interviews. Participation was entirely voluntary, and participants had the right to withdraw at any time without any penalties. Collected data were only accessible to the researcher, independent coder and the two study supervisors.

### Trustworthiness

Guba’s model (Lincoln & Guba 1985:290) of trustworthiness was adhered to in this study. Four important aspects were thus applied throughout the study in dealing with trustworthiness in the qualitative paradigm, namely credibility, transferability, dependability and confirmability. Credibility was achieved through prolonged engagement in the data collection process and enhanced triangulation (Polit & Beck 2017:599). The researcher used telephonic, in-depth interviews and field notes to promote the study’s credibility. Member-checking also occurred when the researcher validated and clarified all interpretations with the participants.

Transferability was ensured through data saturation, purposive sampling and a dense description of the findings with direct quotations from the participants’ interviews. A clear and detailed demographic description of the selected samples was provided to transfer the findings to similar contexts. The researcher also triangulated multiple sources of data.

Dependability was enhanced by means of a dense description of the research methodology, where all aspects of the research were fully described. The researcher conducted in-depth, phenomenological, individual interviews and ensured an audit trail by audio-recording the interviews. The interviews were also coded and recoded.

Confirmability was achieved by presenting an audit trail of the methods, data and analytical processes. It entailed decisions subject to external audit by a reviewer introduced towards the end of the study. The transcripts and audio recordings will be kept for two years after the study’s publication as proof of the chain of evidence.

### Ethical considerations

The researcher obtained approval from the Research Ethics Committee of the Faculty of Health Sciences of the University of Johannesburg (reference number REC-671-2020), and a mental health institution in Limpopo of a higher education institution. Moreover, human participants were involved in the research, and as such, their rights were protected; ethical principles were thus identified and adhered to throughout the study. Dhai and McQuoid-Mason (2011:134) indicated that there are four principles to be considered when conducting research, namely autonomy, nonmaleficence, beneficence and justice. In this study, all these principles were adhered to throughout the research process.

## Results

### Description of participants

Eight participants with relatives diagnosed with SIPD participated in the study. Participants were between the ages of 39 and 68 years. In addition, the years of family members caring for a relative were between 6 and 26 years. They were all black South African men and women who met the sampling criteria. Telephonic interviews lasted 40–60 min each and were conducted in Sepedi. Direct quotations were later translated into English (Selotole 2022:50). The participants’ demographics are presented in Table 1.

**TABLE 1:** Participants’ demographics.

Participant	Gender	Age	Years of caring for a relative	Marital status	Relationship to relative with SIPD
Participant 1	Female	43	6	Cohabitating	Girlfriend
Participant 2	Female	67	20	Widow	Mother
Participant 3	Female	68	17	Single	Sister-in-law
Participant 4	Female	39	8	Single	Sister-in law
Participant 5	Female	54	12	Widow	Mother
Participant 6	Female	49	10	Married	Mother
Participant 7	Female	53	13	Married	Mother
Participant 8	Male	67	26	Divorced	Father

*Source*: Adapted from Selotole, M.S., 2022, *Experiences of family members caring for a relative with substance induced psychosis disorder*, Unpublished Masters Thesis, University of Johannesburg, Johannesburg

SIPD, substance-induced psychosis disorder.

### Presentation of the findings

Three themes emerged from the data analysis, as presented in Table 2.

**TABLE 2:** Themes of the experiences of family members caring for a relative with substance-induced psychosis disorder.

Theme	Catergories
Theme 1	Experienced caring for a relative with SIPD as a destabilising responsibility.
Theme 2	Experienced acceptance and support from significant others and the community and solace in prayer.
Theme 3	Experienced a need for support from government structures in order to care for a relative with SIPD.

*Source*: Adapted from Selotole, M.S., 2022, *Experiences of family members caring for a relative with substance induced psychosis disorder*, Unpublished Masters Thesis, University of Johannesburg, Johannesburg

SIPD, substance-induced psychosis disorder.

#### Theme 1: Experienced caring for a relative with substance-induced psychosis disorder as a destabilising responsibility

From the eight participants’ interviews, it was clear that family members experienced emotional, physical and psychological destabilisation because of the shift in mental health treatment philosophy from institutions to their homes. This situation had a profound impact on their daily routine. Family members shared feelings of helplessness and frustration. They also reported experiencing disappointment because of their unmet expectations for their relatives and feared their relatives’ aggressive nature (Selotole 2022:49).

The following quotations supported these findings:

‘I have mix feelings you know … It is very sad because I am a woman and he is a male, I am scared of him.’ (Participant 5, 54 years, mother)

‘We are trying everything possible to assist him but it seems he doesn’t want to change.’ (Participant 2, 67 years, mother)

‘It is long we have being going up and down with him and no changes. Money is finished.’ (Participant 1, 43 years, girlfriend)

‘I become helpless, only get peace of mind when he is in the hospital … I get frustrated because sometimes people come and claim their staff that he took from them and shout at me.’ (Participant 5, 54 years, mother)

‘[…*W*]e find it is very much frustrating. We don’t know what we can do to assist him. It’s all problems here at home because we can’t do anything for ourselves.’ (Participant 8, 67 years, father)

‘I had big dreams about him, but everything became disappointing and frustrating for me.’ (Participant 1, 43 years, girlfriend)

‘Eish … I feel very sad and discouraged, I was expecting more from him because he is so intelligence such that he passed grade 12, now he changed a family life routine because we must care for him more than ourselves and it is tiring.’ (Participant 5, 54 years, mother)

Family members experienced financial constraints because of their caregiving role, which was a concern for almost all the participants. Family members also said they experienced a lack of social and financial support from the community and their extended family members:

‘Social grant money that he receive is too small for the family because I also not working, I am just sitting here at home looking after him. R1700 is too small, because we buy food, clothes and soaps, this is poverty I am telling you. The five of us depend on it in this household.’ (Participant 8, 67 years, father)

‘At times he demand his grant money (R1700) and burn it with fire. The whole month we survive by begging. It is difficult, it is painful I am telling you.’ (Participant 4, 39 years, sister-in-law)

‘I paid Sangomas a lot of money with no success. The money that I used was the little money that was left by his late father. It is now finished because I was hoping that he will get better one day.’ (Participant 5, 54 years, mother)

Family members experienced social disequilibrium in the community; disequilibrium refers to imbalances in the internal and external environment. According to Fritz et al. (2020:109), family members caring for a relative suffering from SIPD are expected to ensure they balance the internal and external forces in caring for their relatives. Participants reported their experiences as follows:

‘The community also feel that he must be chased away because he and his gangsters are troubling the community at night.’ (Participant 8, 67 years, father)

‘The problem is that when his money is finish he start to demand the other one from his mother by force. If he is not given money he start to be violent towards her. She always make sure she balance her finances to avoid him.’ (Participant 4, 39 years, sister-in-law)

‘His behaviour destroyed our relationship because we don’t talk to one another … Nothing is going well here at home. I really don’t know why all this bad luck.’ (Participants 3, 68 years, sister-in-law)

‘Our mother goes to an extend of borrowing money from shark loans in order to give him whenever he demands it.’ (Participants 2, 67 years, mother)

Family members mentioned that their relatives caused trouble at home and in the community, especially when they relapsed. Some family members reported their experiences as follows:

‘I had problems with him because he didn’t want to know whether is night or day if he want money to buy substances he demand it by force. For me to can be able to sleep I use to make a means and give him. Sometimes when I have money I used to give him before he demands. The problem is that when he finish his money he demands another one and if I don’t give him he start assaulting me and I don’t know what is the problem.’ (Participant 7, 53 years, mother)

‘His case was dismissed by court of law, but I am still paying the damages he have made to the community because I love him and I don’t want to see him in trouble. The problem is I dont have money. ’(Participant 3, 68 years, sister-in-law)

‘The community is very angry with us, especially the daughter of our neighbour. She also threaten to open another police case against us. When he insult them, she also shout back and call us names as well. I also think of leaving this community.’ (Participant 2, 67 years, mother)

#### Theme 2: Experienced acceptance and support from significant others and the community and solace in prayer

Coping strategies are an important aspect of caring for a relative with SIPD. In this study, participants reportedly used some ineffective coping strategies when managing their relative’s demands and disruptive behaviours (Selotole 2022:52). Acceptance was the core strategy family members employed to cope with their caring role. Family members relied on support from community members and ultimately found comfort in prayer, as evidenced by the following quotations:

‘We accepted his condition and his siblings love him as well.’ (Participant 5, 54 years, mother)

‘As a family we accepted this condition, we don’t have problem with him.’ (Participant 4, 39 years, sister-in-law)

‘If I can tell you the truth, here at home we accepted that his condition is not going to be healed, is for a life time. We understand that for him to be better he need support from the family.’ (Participant 7, 43 years, mother)

‘I accepted this condition, I put everything in the hands of God.’ (Participant 1, 43 years, girlfriend)

‘If it was not because of his Uncle’s present where was I going to be as a woman, he is a pillar of my strength.’ (Participant 2, 54 years, mother)

‘His siblings have accepted him here at home and also what he is doing, because they understand that he is suffering from substance-induced psychosis disorder.’ (Participant 7, 43 years, mother)

During the interviews, it became evident that the community and other family members were a primary support system for individuals with a relative diagnosed with SIPD. Some family members narrated:

‘I thank my neighbour. He assist us a lot when my bother relapsed.’ (Participant 4, 39 years, sister-in-law)

‘His uncle is the one who support me when I am in trouble. He doesn’t stay far from us.’ (Participant 3, 68 years, sister-in-law)

‘His uncle always come to my rescue when I am in trouble. He is not staying far from us. When I hear the community saying that he undressed himself, he is doing this and that I call him and he arrive immediately. They have a good relationship. When he relapse I rely on his Uncle for safety.’ (Participant 5, 54 years, mother)

The researcher also noted that acceptance and prayer were amongst the most frequently mentioned coping strategies participants used (Selotole 2022:52). In most interviews, family members shared:

‘I know that prayer have power, myself I don’t have a plan, I believe in prayer you person of God. I know that prayer is powerful, that is why I believe in God. If it was not like that I was going to be attacked by Hypertension.’ (Participant 1, 43 years, girlfriend)

‘This child is my gift from God, I must do everything within my power to take care of him forever. I must pray for him.’ (Participant 6, 49 years, mother)

#### Theme 3: Experienced a need for support from government structures in order to care for a relative with substance-induced psychosis disorder

Family members reported being overwhelmed by their caring role and required government interventions in providing support to SIPD individuals. They spoke about support in terms of finances and housing, because financial strain added to the stress they faced since their relative had been diagnosed with SIPD (Selotole 2022:57). Participants reported:

‘If government can give this patients permanent disability grant it can assist them to better their lives, I finish like that. It is difficult to look after this people without money. Remember everything want money so that life must be easy and we can be able to fulfil our needs and their needs with this little grant.’ (Participant 7, 53 years, mother)

‘I think if government can provide them with permanent disability grant or food parcels so that they can support themselves can be better, because they can’t do anything for themselves in life.’ (Participant 3, 68 years, sister-in-law)

Participants further narrated their experiences about housing as follows:

‘I suggest that all substance-induced psychosis disorder must be supported in the community by government giving them houses. I think in that way government will be giving something best back to the community in supporting substance-induced psychosis disorder patients. People must stop stigmatising us because this is not a personal choice, it just happened and it can happen to everyone and we hope that government can listen to us.’ (Participant 2, 67 years, mother)

‘If government can build *RDP* houses for us, we will see how important they are in the community because this patient belongs to us and government.’ (Participant 4, 39 years, sister-in-law)

During the interviews, most family members caring for a relative with SIPD stated that they did not know how to manage their relatives at home. They consequently suggested that the government build rehabilitation centres to relieve them of their caring roles. Rehabilitation centres will also be beneficial for their relatives suffering from SIPD. This was evidenced by the following quotations:

‘I think if government can build them rehabilitation centres where they can stay and be taught life orientation skills and handcraft work it can be better.’ (Participant 3, 39 years, sister-in-law)

‘If possible government must built them halfway houses where this patients can stay there and not come back home, because we are failing.’ (Participant 4, 39 years, sister-in-law)

‘Limpopo doesn’t have rehabilitation centres where this people can be admitted there and be taught life skills. If government can build rehabilitation centres in our province it will assist in rehabilitation and healing of this people. Look I am not working and he is not working, what do you expect.’ (Participant 8, 67 years, father)

## Discussion of findings

The main objective of this study was to explore and describe family members’ experiences caring for a relative with SIPD. Three themes emerged from the findings of this study, namely that they experienced caring for a relative with SIPD as a destabilising responsibility; they experienced acceptance and support from significant others and the community and solace in prayer; and they experienced a need for support from government structures in order to care for a relative with SIPD. The study’s findings provided insight into family members’ experiences caring for a relative with SIPD. A vital theme was that the family caregivers experienced challenges with caregiving, but they also mentioned changed perspectives regarding caregiving.

### Experienced caring for a relative with substance-induced psychosis disorder as a destabilising responsibility

Participants in this study expressed that they tried everything possible to assist their relatives who have SIPD but became helpless and frustrated when their relatives with SIPD did not want to change. This concurs with a study by Baradwaj, Navin and Kupilli (2018:328), who stated that every family has a dream and high hopes about their relatives’ future. It is thus sad and emotionally destabilising for family members when their relatives do not meet these expectations, especially when they start abusing substances and fail to achieve their future desired goals. Adams (2017:228) reports that unmet expectations cause disappointment and devastation amongst family members caring for a relative with SIPD. In addition, caregiving is associated with a range of emotional, physical and psychological symptoms (George & Vaccarino 2015:319). Caregivers bear the brunt of caring for these relatives, and the situation has worsened since the inception of deinstitutionalisation, as more patients are discharged into the community to be cared for by their families. Moreover, Roberts et al. (2018:63) determined that family members lack experience in caring for a relative diagnosed with SIPD at home. Preston and Epstein (2019:123) similarly concur that the shift in the mental health treatment philosophy from mental health institutions to home-based care had an impact on families’ daily routines.

Participants in this study shared financial difficulties, with one family member expressing that their relative would demand his disability grant and then burn it in the fire, leaving them with no financial assistance for the rest of the month. Baradwaj et al. (2018:44) reported that family caregivers feel helpless and devastated when they care for relatives, especially when their relatives relapse and become aggressive. This relates not only to basic knowledge of caregiving but also to feelings of helplessness and devastation (American Psychiatric Association 2018:387). In addition, Reddy et al. (2016:33) confirmed that relatives with SIPD often sold appliances and various other items from their homes to finance their addiction. This made family members feel helpless, frustrated and confused regarding what to do about their relatives diagnosed with SIPD. Reddy et al. (2016:36) further reported that financial constraints were a concern for almost all family members caring for a relative with SIPD, and Mauri et al. (2017:117) agreed financial constraints were reported to be a major challenge for most individuals providing support to relatives with SIPD worldwide.

### Experienced acceptance and support from significant others and the community and solace in prayer

Participants in this study shared that they ultimately started to accept their relative’s condition and realised it is a lifelong problem. They relied on support from community members and found comfort in prayer. Roberts et al. (2018:489) concurred that acceptance and prayer are the most powerful strategies for family members caring for a relative diagnosed with SIPD. No matter how terrible a situation may be, the first step to improving a bad situation is acknowledging it (Prestley et al. 2018:128). However, Wilson et al. (2018:187) state that disbelief and denial were the first to appear amongst family members caring for a relative suffering from SIPD, followed by blame and anger when a chronic disease such as SIPD was diagnosed. When a relative becomes ill with a brain disorder like SIPD or schizophrenia, feelings of disbelief, sadness and anger are thus common for most caregivers. The last reaction to appear is acceptance, which occurs after family members realise they cannot change the situation (Krause Huhn et al. 2019:178). Wilson et al. (2018:193) further stated that accepting the reality of SIPD as a lifetime challenge does not reduce frustrations but assists in absorbing pressure.

As a result of ineffective coping skills, the researchers observed that family members caring for a relative with SIPD needed support from relatives and the community. Ntescu (2019:33) stresses that family members are the basic support system for a relative with SIPD, and their inability to ineffectively cope with caregiving could lead to emotional distress. However, participants were also able to identify positive aspects of caregiving, especially after being empowered and supported by their relatives. Some family members confirmed that they received support from extended family and neighbours during trying times, but it was insufficient, and they were overwhelmed by their caring role. Similarly, Wisdom, Manuel and Drake’s (2018:123) meta-synthesis illustrated that it is necessary to facilitate the mental health of family caregivers. As mentioned, caregivers seldom receive sufficient help in managing the emotional demands of caregiving.

O’Connell et al. (2019:199) claim the strategic management of SIPD requires joint and consultative efforts between family members, the community, relatives and health professionals for the speedy recovery of individuals diagnosed with SIPD, cared for by relatives at home. Family members also find comfort in prayer as a coping mechanism. In this study, family members reported finding solace in their religious beliefs. Middleton (2020:231) agreed that acceptance and prayer are amongst the most frequently mentioned coping strategies for family members caring for a relative with SIPD.

### Experienced a need for support from government structures in order to care for a relative with substance-induced psychosis disorder

The WHO (2020:330) stated that some family members caring for a relative with SIPD depended on child support grants and disability grants, and they had no other source of income. Participants in this study sometimes went as far as borrowing money from loan sharks so that they could give their relatives whatever they demanded. This is supported by a study conducted by Modise, Mokgaola and Sehularo (2021:6); the researchers stated that participants reported that their salary was not enough to take care of the basic needs of the mental healthcare user (MHCU).

Furthermore, family members also need support from government structures to care for a relative with SIPD at home. Stuart et al. (2014:129) agreed that the government plays a significant role in providing financial support to family members who have relatives diagnosed with SIPD, because financial strain adds to these individuals’ stress levels. Dhai and McQuiod-Mason (2011:45) also reported that proper living environments are part of everyone’s basic needs, regardless of illness. However, family caregivers often live in shambles because when their relative relapses, they vandalise their homes, break windows or burn their rooms (Baradwaj et al. 2018:340). Consequently, family members need support from government structures to care for a relative with SIPD in a proper and dignified manner, especially after discharge (Tylor 2018:123).

A majority of family members caring for a relative with SIPD have no knowledge of how to manage their relatives at home. The WHO (2020:128) thus suggested that governments build rehabilitation centres to relieve family members of their caring role; during relapse, they could utilise these centres. Ultimately, statistics confirmed there are no rehabilitation centres in Limpopo province and only one nongovernmental organisation (halfway house) in Giyani that was closed because of the financial crisis.

The Reconstruction and Development Programme (RDP) is a South African policy framework implemented by the African National Congress (ANC)-led government to address the country’s infrastructure problems. Overall, evidence has proven that 30% – 40% of the South African population has benefited from government schemes since 1994, including people living with disabilities and SIPD (Constitution of RSA 1996:44). Moreover, the WHO (2020:189) reported that SIPD was amongst the conditions that require a significant portion of the mental healthcare budget in the country and worldwide. However, little research has been conducted on this topic, especially in rural areas. According to Mauri et al. (2017:58), further research is required to flatten the curve of SIPD and mental illness worldwide.

### Limitations of the study

Participants in this study were mostly women, which can be an indication that women were more affected by the caregiving role. The researcher also had challenges finding participants to be included in the study, as most caregivers gave excuses when they failed to honour their telephonic appointments. The COVID-19 regulations also impacted the study’s progress because they changed the study’s dynamics from a field study to telephonic interviews; therefore, poor networks and load shedding affected the researcher’s progress.

The researcher had challenges receiving approval from the Limpopo Department of Health to conduct the research, which led to delays in completing the study. Some families were also not comfortable being audio-recorded as they feared their recordings might end up on social media, exposing their situation to the public. Other family members turned down participation at the last minute because of uncertainties.

### Recommendations

It was clear that family members caring for a relative with SIPD needed professional help and support. Psychiatric nurse practitioners play an important role as they come in close contact with family members and their relatives with SIPD. Therefore, they are able to assess the family members’ needs and support them. Psychiatric nurse practitioners should address family members’ feelings of incompetence and strengthen those families who are competent in providing care for their relatives with SIPD. This can be enacted by offering community health education according to family members’ needs. Psychiatric nurse practitioners should also take responsibility for educating the community about mental health by offering mental health awareness campaigns.

The nursing curriculum at training schools should include topics such as family members’ experiences caring for a relative with SIPD and the condition’s effect on family members. Evidence shows there is a need for specialised training when it comes to psychiatric nurses working in mental health settings. Training on SIPD is critical for those working with SIPD because of the complex nature of the condition and potential complications. It was clear from the findings that there was insufficient published research about family members’ experiences caring for a relative with SIPD in Giyani, Limpopo province. Therefore, it is recommended that more research be conducted on SIPD, because combined or integrated interventions were found to be limited. Future research must therefore pay attention to finding ways to improve family involvement in caring for relatives diagnosed with SIPD.

## Conclusion

Generally, it is well known that South Africa has limited mental health and community support available for patients with mental illnesses and their caregivers because of stigmatisation. Mental healthcare professionals and the Mental Health Directorate need to introduce suitable mental health interventions such as group therapy, family home visits and family therapy to assist the family members caring for relatives with SIPD in developing healthy coping strategies.
